# S252. HEALTH CARE RESOURCE UTILISATION IS HIGHER IN PATIENTS PRIOR TO DIAGNOSIS WITH SCHIZOPHRENIA THAN NON-SCHIZOPHRENIA COMPARATORS IN A LARGE COMMERCIALLY-INSURED POPULATION IN THE UNITED STATES

**DOI:** 10.1093/schbul/sby018.1039

**Published:** 2018-04-01

**Authors:** Anna Wallace, Keith Isenberg, John Barron, Whitney York, Mayura Shinde, Matt Sidovar, Jessica Franchino-Elder, Michael Sand

**Affiliations:** 1 Healthcore, Inc; 2 Anthem Inc; 3 Health Economics and Outcomes Research, Boehringer Ingelheim Pharmaceuticals, Inc; 4 Boehringer Ingelheim Pharma GmbH & Co. KG

## Abstract

**Background:**

Schizophrenia is associated with considerable health care resource utilisation (HCRU) and costs, yet little is known about the patterns of care and HRCU in patients with schizophrenia prior to diagnosis. To address this knowledge gap, we examined the HCRU of patients with and without schizophrenia over a 5-year pre-diagnosis period.

**Methods:**

This US-based retrospective study used claims data from the HealthCore Integrated Research Database to identify newly diagnosed patients with schizophrenia (ICD-9: 295.x, ICD-10: F20.x) aged 15–54 years at diagnosis. Patients with schizophrenia were compared with a demographically matched (1:4) non-schizophrenia cohort during the 0–12 months, >1–2, >2–3, >3–4 and >4–5 years prior to schizophrenia diagnosis. During the pre-diagnosis periods, both all-cause and behavioural health-related HCRU were described.

**Results:**

The schizophrenia and comparator cohorts included 6,732 and 26,928 patients, respectively. The most common types of schizophrenia were schizoaffective disorder (49%), paranoid (24%) and unspecified (19%). Patients were distributed across all major US regions (Northeast: 18%, Midwest: 27%, South: 29%, West: 27%). Average age at diagnosis was 32.8 years and most patients were male (57.4%). The percentage of patients with at least one all-cause inpatient hospitalisation in the 0–12 months prior to diagnosis was 32.7% for patients with schizophrenia versus 3.9% for comparators. Patients with schizophrenia had a greater mean number of all-cause physician office visits in all pr- diagnosis time periods versus comparators (schizophrenia: 4.5–5.5 visits, comparators: 3.1–3.2 visits). Behavioural health-related HCRU was also more substantial in patients with schizophrenia versus comparators across all time periods in terms of the mean number of visits to a psychiatrist (1.8–2.9 vs 0.1 visits, respectively) or a psychologist (1.0–1.2 vs 0.2 visits, respectively). The percentage of patients with claims for antipsychotic medication was also greater in the schizophrenia cohort vs comparators (21.8–56.6% vs 0.7–1.0% of patients, respectively).

**Discussion:**

For up to 5 years prior to diagnosis, patients with schizophrenia have higher all-cause and behavioural health-related HCRU, in addition to higher use of anti-psychotic medications, compared with matched comparators. In the schizophrenia cohort, HCRU increased in frequency closer to diagnosis, compared with matched comparators, whose HCRU remained relatively stable.

This study improves our understanding of the characteristics of clinically high-risk patients who go on to develop schizophrenia, who have more frequent encounters with health care providers than comparators. These results also suggest that early identification and treatment of patients prior to schizophrenia diagnosis could be optimised and is warranted.

Funding:

Boehringer Ingelheim (ANTHEM)

